# Universal, school-based, interventions to improve emotional outcomes in children and young people: a systematic review and meta-analysis

**DOI:** 10.3389/frcha.2025.1526840

**Published:** 2025-06-02

**Authors:** Daniel Hayes, Emre Deniz, Kirsty Nisbet, Abigail Thompson, Anna March, Carla Mason, Joao Santos, Rosie Mansfield, Emma Ashworth, Bettina Moltrect, Shaun Liverpool, Hannah Merrick, Jan Boehnke, Neil Humphrey, Paul Stallard, Praveetha Patalay, Jessica Deighton

**Affiliations:** ^1^ Evidence Based Practice Unit, Anna Freud National Centre for Children, and Families, London, United Kingdom; ^2^ Department of Behavioural Science and Health, University College London, London, United Kingdom; ^3^ Department of Education, University of York, York, United Kingdom; ^4^ Department of Public Health and Sport Sciences, Exeter Medical School, University of Exeter, Exeter, United Kingdom; ^5^ Manchester Institute of Education, University of Manchester, Manchester, United Kingdom; ^6^ Centre for Longitudinal Studies, Social Research Institute, University College London, London, United Kingdom; ^7^ School of Psychology, Liverpool John Moores University, Liverpool, United Kingdom; ^8^ Department of Allied Health, Social Work and Wellbeing, Edge Hill University, Ormskirk, United Kingdom; ^9^ Population Health Sciences Institute, Newcastle University, Newcastle upon Tyne, United Kingdom; ^10^ School of Health Sciences, University of Dundee, Dundee, United Kingdom; ^11^ Department for Health, University of Bath, Bath, United Kingdom; ^12^ MRC Unit for Lifelong Health and Ageing, Population Science and Experimental Medicine, University College London, London, United Kingdom; ^13^ Department for Clinical, Educational and Health Psychology, University College London, London, United Kingdom

**Keywords:** school, universal, mental health, pupil, emotional

## Abstract

**Introduction:**

There is debate into the impact of universal, school-based interventions to improve emotional outcomes. Previous reviews have only focused on anxiety and depression symptoms, omitting broader internalising symptoms, nor include the proliferation of newer studies which have focused on mindfulness in schools.

**Methods:**

We conducted a systematic review and meta-analysis, searching MEDLINE, Embase, PsycINFO, and Cochrane Central Register of Controlled trials for studies focusing on universal interventions to improve emotional outcomes for young people aged 8–18 until 15/12/2022. The primary focus were post-intervention self-report anxiety, depression and internalising outcomes. We prospectively registered the study with PROSPERO, number (CRD42020189845). Risk of bias was assessed using specially devised tools adopted from Cochrane.

**Results:**

In total, 71 unique studies with a total sample of 63,041 young people met the inclusion criteria. This included 40 studies with 35,559 participants for anxiety outcomes, 50 studies with 49,418 participants for depression outcomes, and 15 studies with 21,473 participants for internalising outcomes. Pupils who received universal school-based interventions had significantly improved anxiety (d = −0.0858, CI = −0.15, −0.02, z = −2.46, *p* < .01) and depression (d = −0.109, CI = −0.19, −0.03, z = −2.60, *p* < 0.013), but not internalising outcomes. For anxiety disorders, intervention theory moderated the intervention effectiveness (Q = 24.93, *p* < 0.001), with CBT principles being significantly more effective than those that applied mindfulness or other/multiple theories.

**Discussion:**

Evidence suggests that universal, school-based approaches for anxiety and depression produce small effect sizes for pupils. We conclude that used as a population health approach, these can have an impactful change on preventing anxiety and depression. However, intervention developers and researchers should critically consider which theories/approaches are being applied, particularly when trying to improve anxiety outcomes.

**Systematic Review Registration:**

PROSPERO CRD42020189845.

## Introduction

Epidemiological rates of mental health difficulties in children and young people range between 10% and 20%, with emotional difficulties such as anxiety and depression being the most prevalent (1, 2). Depressive disorders are the third most frequent cause of adolescent disability-adjusted life-years lost, whilst anxiety disorders rank third among the causes of adolescent disability-adjusted life-years lost in High Income Countries (3). With increasing numbers of young people being affected by mental health problems, and international data indicating that more than 60% of those in need do not have access to adequate treatment, youth mental health has become a major public health concern (4). Without input, difficulties can have a significant detrimental effect on physical, social and psychological outcomes in adulthood (5, 6).

Youth mental health services have been experiencing a shift in recent years, putting a greater focus on schools as key providers of mental health provision (7, 8). Considering the amount of time that children and young people spend at school and the existing infrastructure to deliver intervention programmes, schools can be an important setting to deliver different mental health interventions (9, 10). Furthermore, research suggests that school-based mental health provision helps overcome important social and environmental barriers to accessing support, including transport costs, social stigma or family-related factors (11, 12).

School-based interventions have been broadly classified into promotion, prevention or treatment approaches. Promotion programmes aim to proactively increase young people's wellbeing by fostering strengths and competences (13). Preventative interventions primarily aim to prevent mental health problems from arising by targeting known risk and protective factors (14). Interventions in the treatment category address existing difficulties by assessing symptoms and specifically treating them. Furthermore, school interventions can either follow a universal approach being delivered to all pupils, or they are designed as targeted interventions, implemented with specific individuals with known risk factors or already displaying difficulties.

In the UK, a 2013 national survey of schools suggested a clear trend towards reactive interventions, with 71.2% of secondary schools implementing interventions due to children in their school starting to show symptoms or already experiencing some form of mental health problem (15). While universal prevention and promotion interventions offer a number of advantages, including being sensitive to emotional disorders that may develop later in life, being destigmatising, reaching a wide range of children, being cost and time effective, and promoting adaptive coping/resilience across an array of experiences and settings, they have traditionally been underused and undervalued relative to other types of interventions.

More recently, there has been a shift towards the use of universal whole-school prevention interventions (16, 17). By introducing early intervention for all pupils, it is thought that we can effectively “immunise” them from later difficulties (15). This avoids costly screening procedures needed to identify those at-risk, prevents the issue of some at-risk children being missed, and removes the need for the highly trained professionals often required to deliver targeted interventions (18).

Notably, evidence of existing interventions to prevent emotional outcomes such as depression and anxiety symptoms in youth have been mixed. Many previous reviews (19–21) of school-based prevention interventions have found small or modest effect size for anxiety and depressive outcomes which last up to 12 months post intervention. However, a 2019 meta-analysis (14) and corresponding NIHR report (22) concluded that overall, there was limited evidence of universal interventions in schools for reducing depression or anxiety symptoms. Specifically, these studies concluded that in primary school settings, there was weak evidence to suggest interventions incorporating cognitive behavioural therapy (CBT) reduced anxiety symptoms. Whilst in secondary school settings, there was some evidence to suggest mindfulness/relaxation and cognitive behavioural therapy (CBT) reduced anxiety symptoms.

Some limitations exist when interpreting previous findings. Firstly, studies with very small sample sizes (i.e., less than 32 participants per arm) were included (14, 22) which is vulnerable to Type I and Type II errors due to lack of statistical power (23). Secondly, most reviews of interventions for emotional difficulties only include studies utilising measures of anxiety and depression symptoms to determine the effectiveness of an intervention (24). This means that interventions that target wider constructs for emotional difficulties have not adequately been examined and so their effectiveness is not established. Lastly, conclusions about mindfulness interventions have been based on a small number of studies (*n* = 3). Since these reviews, a number of high-profile studies focusing on this topic area have been published. This warrants further investigation given the increasing interest and rollout of mindfulness in schools to support mental health. In light of these points, we aimed to further investigate the impact of universal, school-based interventions on emotional difficulties in pupils.

## Materials and methods

### Search strategy and inclusion criteria

For this review and meta-analysis, we developed a search strategy mapping to the PICO criteria (S0) and searched MEDLINE, Embase, PsycINFO, and Cochrane Central Register of Controlled trials for studies published until 15th December 2022. A detailed search strategy is available in the Supplementary Materials S1, as are definitions for examined constructs (S2). Hand searching of included articles and consultation with experts (*n* = 9) was also undertaken.

We included studies if they were randomised or quasi-randomised trials of school-based, universal interventions targeting emotional outcomes; anxiety, depression, or internalising symptoms, for young people aged 8–18 years old. This age range was selected to reflect the ages of pupils who could self-report their difficulties. Randomisation could occur at individual and/or class level. We also excluded studies where there were less than 32 participants in at least one arm, as this is needed to detect a one standard deviation difference in improvement with adequate statistical power (80%) and a significance level of 0.05 (25). There were no exclusions on the type, format of intervention delivery method. Searches were restricted to those in English.

We screened articles in two stages. Both first and second stage screening were double screened by at least two researchers (DH, ED, KN, AT, CM, AM, JS, RM, HM, JD) and any disagreements were resolved by a third reviewer. The lead reviewer of this article (DH) checked a 10% sample of records of other reviewer dyads to ensure consistency across screening. We employed a uniform approach to data extraction, using a developed data extraction template (see Supplementary Materials S3) which focused on bibliographic information (e.g., study year), school characteristics, measures used (e.g., name, as primary/secondary outcome), intervention characteristics (e.g., length, theoretical underpinning), and information for the meta-analysis (e.g., means, standard deviations, sample size). When articles used both anxiety and depression measures and there was no information as to whether these were primary or secondary outcomes, we used the first listed measure as the primary. Data extraction was undertaken by one of the researchers previously involved in screening and checked by DH and ED.

### Quality appraisal

Methodological quality of the included studies was assessed by four researchers (DH, ED, KN and AT), independently, using two specially devised risk of bias tools adapted from Cochrane Risk of Bias Tool for Randomized Trials (26) and Cochrane Risk of Bias Tool in Non-randomized Studies of Interventions’ (27). These tools have previously been adopted by other researchers (28). Quality appraisal were judged based on risk of bias due to: (i) randomisation (RCT) or confounding variables (QED), (ii) deviations from the intended interventions, (iii) missing outcome data, (iv) measurement in outcomes, and (v) selection of reported results. Based on the risk of bias tools’ guidelines (26, 27), each study was evaluated and judged on an overall risk of bias score by two researchers, independently assigning one of the following ratings: low risk, some concerns, and high risk. The lead reviewer of this article (DH) checked a 10% sample of records of other reviewer dyads.

### Data analysis

Statistical analyses were performed using R version 4.2.3. Due to the heterogenous nature of the data from the included studies, random effects meta-analyses were reported for outcomes related to anxiety, depression, and internalising outcomes using standardised mean differences (Cohen's d). Additionally, I^2^ statistics were performed to report heterogeneity. In addition, we conducted subgroup analyses to report whether the pooled intervention effects were moderated by certain study or intervention characteristics such as study design, methodological quality, outcome type, intervention duration, interventionist, school type, control condition, and intervention theory. In subgroup analyses, each subgroup was kept at three or lower groups to minimise the potential for false-positive results (29). Finally, studies with no sufficient quantitative data (i.e., post-intervention means and standard deviations) were excluded from the meta-analyses, unless they reported other quantified data that could be used to calculate effect sizes (e.g., standard error, effect size, etc). Funnel plots and Egger's test were used to explore potential publication bias.

### Role of the funding source

The funder of the study had no role in study design, data collection, data analysis, data interpretation, writing the report, or the decision to submit for publication. DH, ED and JD had access to the data in the study. DH and JD had final responsibility for the decision to submit for publication.

## Results

**Figure 1:**
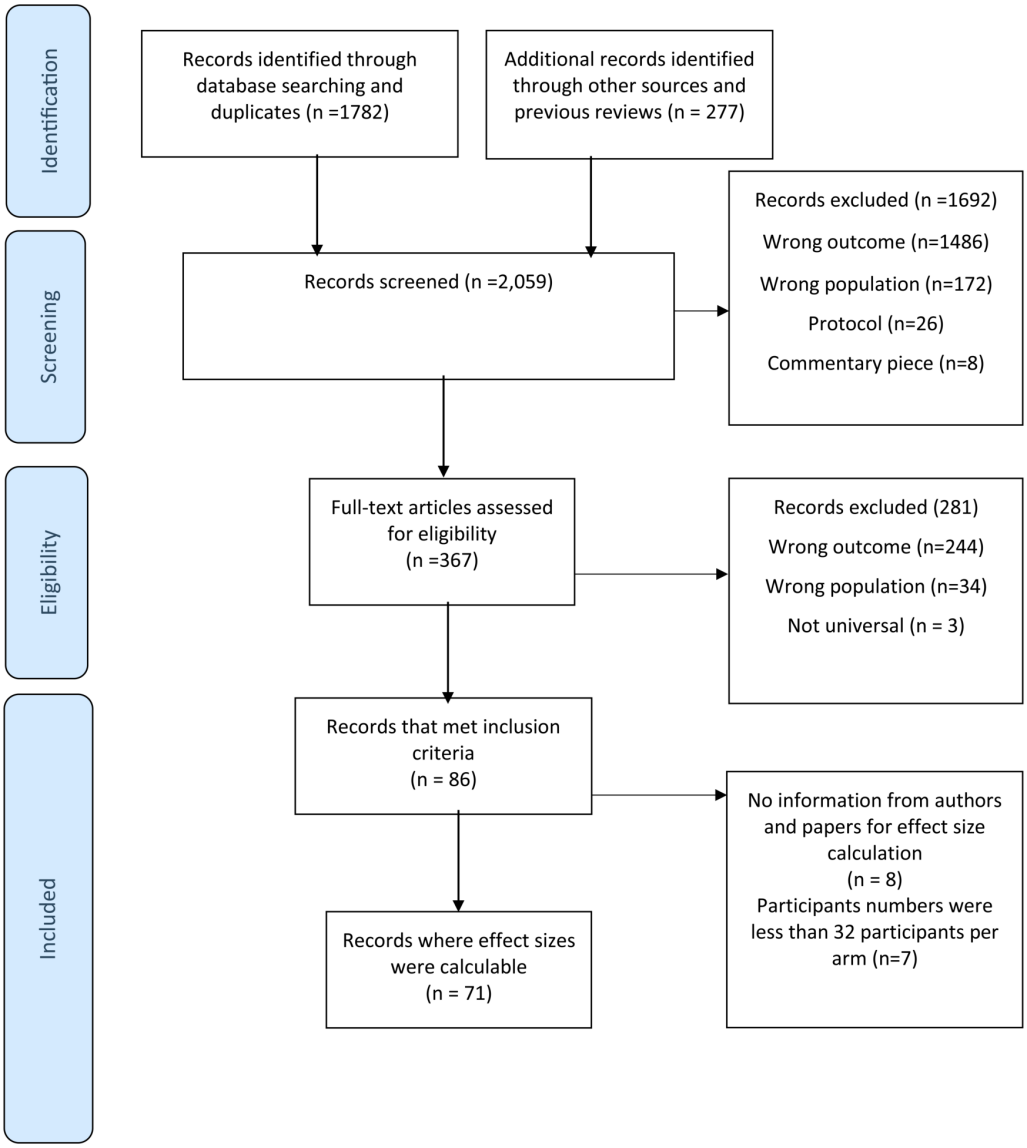
PRISMA flow chart [adapted from Moher et al. (37)].

We screened 2,059 titles and abstracts and 367 full text records (see Figure 1). In total, 71 unique studies with a total sample of 63,041 participants were included. The PRISMA flow chart shows reasons for exclusion at each stage. At both first and second stage screening, the most common reason was the wrong outcomes being studies (*n* = 1,486 and *n* = 244, respectively.

### Study characteristics

Studies were conducted between 1993 and 2022 in 22 different countries. More than half of the included studies were conducted in Australia (*n* = 27) and USA (*n* = 9) and most studies took place in the past decade (*n* = 44). Additionally, the majority of the included studies applied a RCT design (*n* = 60). Included studies were highly heterogeneous in terms of the duration and frequency of the delivered universal interventions, which ranged from a single 30-minute session to 2 h 50 min per week for four school years. Moreover, facilitators of universal interventions also varied across studies, with the majority being delivered by teachers (*n* = 36) followed by a psychologist (*n* = 22). The majority of studies (*n* = 51) were conducted in secondary schools, 19 were conducted in primary schools, and one study did not specify. In all included studies, children/young people reported their own anxiety and depression symptoms (*n* = 64); however, in six studies parents (*n* = 3) or teachers (*n* = 3) were the reporters of their children's internalising symptoms. The content of interventions were highly heterogeneous and included theoretical bases in CBT (*n* = 29), mindfulness (*n* = 11), and either one another, or multiple theories (*n* = 31). The most common intervention package used were alterations of the FRIENDS program for both anxiety (*n* = 11) and depression (*n* = 9). Unbranded (i.e., no named) interventions were most commonly used for internalizing difficulties (*n* = 5) The following were the most commonly used scales to measure children's emotional outcomes: The Spence Children's Anxiety Scale (30), The Children's Depression Inventory (31), and The Strengths and Difficulties Questionnaire for internalising symptoms (32).

In terms of methodological quality, 9 studies showed low risk of bias, while the majority showed some methodological concerns (*n* = 48). 14 studies showed high risk of bias. Study characteristics and corresponding quality appraisals are outlined in Tables 1–3.

**Table 1:** Study characteristics for studies exploring universal school interventions on anxiety symptoms.

Author	Design	Country	Outcome	Length	Intervention name and theory	Deliverer	Training	School	Measure	Control	ROB
1. Ahlen, 2018/ 2019	RCT	Sweden	Primary	600	FRIENDS for life CB	Teacher	Yes	Primary	SCAS	Wait list control	High
2. Andrews, 2021	RCT	Australia	Secondary	250	Climate schools CB	Teacher	Not specified	Secondary	GAD-7	Active control	High
3. Araya, 2013	RCT	Chile	Secondary	660	Yo, Pienso, Siento CB	Facilitator	N/A—professional	Secondary	RCADS	Wait list control	Low
4. Aune, 2009	RCT	Norway	Secondary	135	The Norwegian Universal Preventive Program for Social Anxiety CB	Psychologist	N/A—professional	Secondary	SACRED	No intervention	Some concerns
5. Barrett, 2005	RCT	Australia	Primary	525	FRIENDS CB	Psychologist	N/A—professional	Secondary	SCAS	No intervention	Some concerns
6. Barrett, 2001	RCT	Australia	Primary	750	Friends for children CB	Psychologist	Not specified	Primary	SCAS	No intervention	Some concerns
7. Britton, 2014	RCT	US	Primary	210	No name M	Teacher and self-directed	Yes	Primary	STAI-C	Active control	Some concerns
8. Calear, 2009	RCT	Australia	Primary	275	MoodGYM CB	Teacher	N/A—self directed	Secondary	RCMAS	Wait list control	Low
9. Calear, 2016	RCT	Australia	Primary	210	e-couch anxiety and worry programme multiple	Self-directed	N/A	Secondary	SCAS	Wait list control	Low
10. Challen, 2014	QED	England	Secondary	1080	UK resilience programme multiple	Teacher	Yes	Secondary	RCMAS	No intervention	High
11. Collins, 2014	RCT	Scotland	Primary	550	No name CB	Psychologist	Yes	Primary	SCAS	No intervention	Some concerns
12. Essau, 2012	RCT	Germany	Primary	550	FRIENDS CB	Facilitator	Yes	Secondary	SCAS	Wait list control	Some concerns
13. Frank, 2021	RCT	US	Secondary	660	Learning to breathe M	Teacher	Yes	Secondary	GAD-7	No intervention	Some concerns
14. Gallegos, 2008	RCT	Mexico	Primary	675	FRIENDS for Life CB	Teacher	Yes	Both (mean age primary)	SCAS	No intervention	Some concerns
15. Gaucht, 2017	RCT	Belgium	Primary	480	No name ACT	Teacher	Yes	Secondary	YSR—Anxiety	No intervention	Low
16. Johnson, 2019	QED	Australia	Secondary	120	Mindfulness training for teens M	Facilitator	N/A—professional	Secondary	GAD-7	Wait list control	High
17. Johnson, 2021	RCT	Australia	Secondary	750	Mindfulness training for teens M	Facilitator	N/A—professional	Secondary	GAD-7	No intervention	Some concerns
18. Johnson, 2016	RCT	Australia	Primary	380	.b (Dot be) M	Facilitator	N/A—professional	Secondary	DASS-21	No intervention	Some concerns
19. Johnson, 2017	RCT	Australia	Primary	450	.b (Dot be) M	Facilitator	N/A—professional	Secondary	DASS-21	No intervention	Low
20. Kato, 2017	QED	Japan	Primary	450	Fun FRIENDS CB	Teacher	Not specified	Primary	SCAS	No intervention	Some concerns
21. Khalsa 2011	RCT	US	Secondary	825	Yoga Ed Y	Facilitator	N/A—professional	Secondary	POMS-SF	No intervention	Some concerns
22. Kuyken, 2022	RCT	England	Secondary	400	No name (MYRIAD) M	Teacher	Yes	Secondary	RCADS	No intervention	Some concerns
23. Lock, 2003	RCT	Australia	Primary	1,200	FRIENDS CB	Psychologist	N/A—professional	Secondary	SCAS	Wait list control	Some concerns
24. Lowry-Webster, 2001	RCT	Australia	Primary	600	FRIENDS CB	Teacher	Yes	Secondary	SCAS	Wait list control	High
25. Miller et al., 2011	RCT	Canada	Primary	540	FRIENDS CB	Teacher	Yes	Primary	MASC	Wait list control	Some concerns
26. Perkins, 2020	RCT	England	Secondary	30	No name CB	Unguided	N/A—self directed	Secondary	RCADS	Wait list control	Some concerns
27. Quach, 2016	RCT	US	Secondary	15	No name multiple	Facilitator	N/A—Professional	Secondary	SCARED	Wait list control	Some concerns
28. Rapee, 2020	RCT	Australia	Secondary	600	Friendly schools and cool kids—taking control SE	Teacher	Yes	Primary	SCAS	No intervention	Some concerns
29. Roberts, 2003	RCT	Australia	Secondary	660	Penn prevention program CB	Facilitator	Yes	Secondary	RCMAS	No intervention	Some concerns
30. Roberts, 2010	RCT	Australia	Primary	600	Aussie optimism program CB	Teacher	Yes	Primary	RCMAS	No intervention	High
31. Rooney, 2013	RCT	Australia	Primary	600	Positive thinking skills program CB	Teacher	Yes	Primary	SCAS	No intervention	Some concerns
32. Rooney, 2006	RCT	Australia	Secondary	480	Positive thinking programme CB	Psychologist	Yes	Primary	RCMAS	No intervention	Some concerns
33. Ruttledge, 2016	RCT	Ireland	Primary	550	FRIENDS for Life CB	Teacher	Yes	Primary	SCAS	Wait list control	Some concerns
34. Sheffield, 2006	RCT	Australia	Secondary	380	No name multiple	Teacher	Yes	Secondary	SCAS	No intervention	High
35. Shum, 2019	QED	Hong Kong	Primary	468	The adventures of DoReMiFa multiple	Facilitator	Yes	Primary	SCARED;	No intervention	High
36. Teesson, 2020	RCT	Australia	Primary	240	Climate schools CB	Teacher	Not specified	Secondary	GAD-7;	Active control	Some concerns
37. Tomba, 2010	RCT	Italy	Primary	360	No name Multiple	Psychologist	N/A—professional	Secondary	KSQ	Active control	Some concerns
38. Velásquez, 2015	RCT	Columbia	Primary	2,880	No name Y	Teacher	N/A—professional	Unclear	SDQ	Wait list control	High
39. Venturo-Connerly, 2022	RCT	Kenya	Primary	40	No name PS	Lay	Not specified	Secondary	GAD-7	Active control	Some concerns
40. Wong, 2014	RCT	Australia	Primary	373.75	This way up schools CB	Teacher	None	Secondary	GAD-7	Wait list control	Some concerns

ACT, acceptance and commitment therapy; CB, cognitive behavioural; DASS-21, depression, anxiety, and stress scale; GAD-7, the general anxiety disorder-7; KSQ, Kellner's symptom questionnaire; M, mindfulness; MASC, multidimensional anxiety scale for children; N/A, not applicable; POMS-SF, profile of mood states-short form (POMS-SF); PP, positive psychology; PS, problem solving; QED, quasi experimental design; RCADS, revised children's anxiety and depression scale; RCMAS, revised children's manifest anxiety scale; RCT, randomised controlled trial, SCARED; the screen for child anxiety–related emotional disorders; SACRED, screen for child anxiety-related emotional disorders; SCAS, the spence children's anxiety scale; SDQ, strengths and difficulties questionnaire; SE, social emotional; STAI-C, the Spielberger state-trait anxiety inventory; US, United States; Y, yoga; YSR, the youth self-report questionnaire.

**Table 2:** Study characteristics for studies exploring universal school interventions on depressive symptoms.

Author	Design	Country	Outcome	Length	Intervention name and theory	Deliverer	Training	School	Measure	Control	ROB
1. Ahlen, 2018/2019	RCT	Sweden	Primary	600	FRIENDS for life CB	Teacher	Yes	Primary	CDI	Wait list control	High
2. Andrews, 2021	RCT	Australia	Secondary	250	Climate schools CB	Teacher	Not specified	Secondary	PHQ-9	Active control	High
3. Anttilia, 2021	QED	Finland	Primary	270	DepisNet SDT	Unguided	None	Secondary	RBDI	No intervention	Some concerns
4. Antunes Lima 2022	RCT	Brazil	Primary	5,200	No name Not specified	Teacher	N/A—Professional	Secondary	CES-D	No intervention	Some concerns
5. Araya, 2013	RCT	Chile	Primary	660	Yo, Pienso, Siento CB	Facilitator	N/A—Professional	Secondary	BDI	Wait list control	Low
6. Aune, 2009	RCT	Norway	Secondary	135	The Norwegian Universal Preventive Program for Social Anxiety CB	Psychologist	N/A—Professional	Secondary	SMFQ	No intervention	Some concerns
7. Barrett, 2001	RCT	Australia	Secondary	750	Friends for children CB	Psychologist	Not specified	Primary	CDI	No intervention	Some concerns
8. Calear, 2009	RCT	Australia	Primary	275	MoodGYM CB	Teacher	None	Secondary	CES-D	Wait list control	Low
9. Challen, 2014	QED	England	Primary	1,080	UK resilience programme Multiple	Teacher	Yes	Secondary	CDI	No intervention	High
10. Clarke 1993	RCT	US	Primary	150	No name Not specified	Teacher	Yes	Secondary	CES-D	No intervention	Some concerns
11. Essau, 2012	RCT	Germany	Primary	550	FRIENDS CB	Facilitator	Yes	Secondary	RCADS	Wait list control	Some concerns
12. Gallegos, 2008	RCT	Mexico	Primary	675	FRIENDS for Life CB	Teacher	Yes	Primary and secondary (primary age)	CDI	No intervention	Some concerns
13. Gillham 2007	RCT	US	Primary	1,080	Penn resiliency program Multiple	Teacher or counsellor	Yes	Secondary	CDI	Active control	Some concerns
14. Horowitz, 2007	RCT	US	Primary	720	No name IPP	Psychologist	N/A—Professional	Secondary	CDI	No intervention	Some concerns
15. Johnson, 2021	RCT	Australia	Secondary	750	Mindfulness training for teens M	Facilitator	N/A—Professional	Secondary	DASS-21	No intervention	Some concerns
16. Johnson, 2019	QED	Australia	Secondary	120	Mindfulness training for teens M	Facilitator	N/A—Professional	Secondary	DASS-21	Wait list control	High
17. Johnson, 2016	RCT	Australia	Primary	380	b (Dot be) M	Facilitator	N/A—Professional	Secondary	DASS-21	No intervention	Some concerns
18. Jones 2010	RCT	US	Secondary	1,920	4Rs program multiple	Teacher	Yes	Primary	DISC	Not stated	Low
19. Kato, 2017	QED	Japan	Secondary	450	Fun FRIENDS CB	Teacher	Not specified	Primary	DSDR	No intervention	Some concerns
20. Khalsa 2011	RCT	US	Primary	825	Yoga Ed Y	Facilitator	N/A—Professional	Secondary	POMS-SF	No intervention	Some concerns
21. Kuyken, 2013	QED	England	Secondary	495	MiSP programme M	Teacher	Yes	Secondary	CES-D	No intervention	High
22. Kuyken, 2022	RCT	England	Primary	400	No name (MYRIAD study) M	Teacher	Yes	Secondary	CES-D	No intervention	Some concerns
23. Lock, 2003	RCT	Australia	Secondary	540	FRIENDS CB	Psychologist	N/A- Professional	Primary	CDI	No intervention	Some concerns
24. Lock, 2003b	RCT	Australia	Secondary	1,200	FRIENDS CB	Psychologist	N/A—Professional	Secondary	CDI	Wait list control	Some concerns
25. Lowry-Webster, 2001	RCT	Australia	Secondary	600	FRIENDS CB	Teacher	Yes	Secondary	CDI	Wait list control	High
26. Merry 2004	RCT	New Zealand	Primary	605	RAP-Kiwi CB	Teacher	Yes	Secondary	BDI	Active control	Some concerns
27. Olive, 2019	RCT	Australia	Primary	15,400	No name (LOOK study) PA	Teacher	N/A—professional	Primary	CDI	No intervention (usual practice)	Some concerns
28. Possel, 2005	RCT	Germany	Secondary	901	LISA Multiple	Psychologist	Yes	Secondary	SBB-DES	No intervention	Some concerns
29. Possel, 2011	RCT	Germany	Primary	900	LARS&LISA Multiple	Psychologist	Yes	Secondary	SBB-DES	No intervention	Some concerns
30. Possell, 2013	RCT	US	Primary	900	LARS&LISA CB	Facilitator	N/A—Professional	Secondary	CDI	No intervention	Some concerns
31. Raes 2014	RCT	Belgium	Primary	800	No name M	Facilitator	N/A—Professional	Secondary	DASS-21	No intervention	Some concerns
32. Rapee, 2020	RCT	Australia	Secondary	600	Friendly schools and cool kids—taking control SE	Teacher	Yes	Primary	SMFQ	No intervention	Some concerns
33. Rivet-Duval, 2011	RCT	Mauritius	Primary	660	RAP-A Multiple	Teacher	Yes	Secondary	RADS-2	Waitlist control design.	Some concerns
34. Roberts, 2003	RCT	Australia	Primary	660	Penn Prevention Program CB	Facilitator	Yes	Secondary	CDI	No intervention (usual practice)	Some concerns
35. Roberts, 2010	RCT	Australia	Primary	600	Aussie optimism program CB	Teacher	Yes	Primary	CDI	No intervention	High
36. Rooney, 2013	RCT	Australia	Primary	600	Positive thinking skills program CB	Teacher	Yes	Primary	CDI	No intervention	Some concerns
37. Rooney, 2006	RCT	Australia	Primary	480	Positive thinking programme CB	Psychologist	Yes	Primary	CDI	No intervention	Some concerns
38. Rose, 2014	RCT	Australia	Primary	605	RAP and peer interpersonal relatedness (PIR) program CB + IT	Facilitator	Yes	Secondary	RADS-2	No intervention	Some concerns
39. Sælid, 2022	RCT	Norway	Primary	960	MindPower CB	Teacher	Yes	Secondary	RADS-2	Stepped control	Some concerns
40. Sawyer, 2010	RCT	Australia	Primary	425	Beyondblue CB	Teacher	Yes	Secondary	CES-D	No intervention	High
41. Sheffield, 2006	RCT	Australia	Primary	380	No name CB	Teacher	Yes	Secondary	CDI	No intervention	Some concerns
42. Shochet, 2001	QED	Australia	Primary	495	Resourceful adolescent program Multiple	Psychologist	Yes	Secondary	CDI	Wait list control	Some concerns
43. Tak 2016	RCT	The Netherlands	Primary	800	Op Volle Kracht CB	Psychologist	Yes	Secondary	CDI	No intervention	Some concerns
44. Teesson, 2020	RCT	Australia	Primary	240	Climate schools CB	Teacher	Not specified	Secondary	PHQ	Active control	Some concerns
45. Tomba 2010	RCT	Italy	Primary	360	No name Multiple	Psychologist	N/A—Professional	Secondary	KSQ	Active control	Some concerns
46. Velásquez, 2015	RCT	Columbia	Primary	2,880	No name Y	Teacher	N/A—Professional	Primary and secondary (age not specified)	SDQ	Waitlist control	High
47. Volanen 2020	RCT	Finland	Primary	405	Healthy learning mind M	Facilitator	N/A—Professional	Secondary	BDI	Active control	Some concerns
48. Volkeart, 2022	RCT	Belgium	Secondary	550	Boost camp ER	Psychologist	N/A—Professional	Secondary	CDI	No intervention	Some concerns
49. Wong, 2012	QED	Hong Kong	Primary	630	The little prince is depressed Not specified	Teacher	Yes	Secondary	DASS-21	No intervention	Some concerns
50. Wong, 2014	RCT	Australia	Primary	373.75	This way up Schools CB	Teacher	None	Secondary	PHQ-9	Wait list control	Some concerns

ACT, acceptance and commitment therapy; BDI, beck depression inventory; CB, cognitive behavioural; CDI, the children's depression inventory; CES-D, center for epidemiological studies depression scale; COG, cognitive; DASS-21, depression, anxiety, and stress scale; DISC, diagnostic interview schedule for children predictive scales; ER, emotional regulation; IPP, interpersonal prevention program; IT, interpersonal therapies; KSQ, Kellner's symptom questionnaire; M, mindfulness; N/A, not applicable; PA, physical activity; PHQ-9, patient health questionnaire-9; POMS-SF, profile of mood states-short form (POMS-SF); PP, positive psychology; PS, problem solving; QED, quasi experimental design; RADS-2, Reynolds adolescent depression scale-2; RBDI, Raitasalo depression scale; RCADS, revised children's anxiety and depression scale; RCT, randomised controlled trial; SBB-DES, the self-report questionnaire, depression; SDQ, strengths and difficulties questionnaire; SDT, self- determination theory; SIPM, social information processing model; SMFQ, short mood and feelings questionnaire; SPF, social and protective factors; US, United States; Y, yoga.

**Table 3:** Study characteristics for studies exploring universal school interventions on internalising symptoms.

Author	Design	Country	Outcome	Length	Intervention name and theory	Deliverer	Training	School	Measure	Control	ROB
1. Andrews, 2021	RCT	Australia	Primary	250	Climate schools CB	Teacher	Not specified	Secondary	SDQ	Active control	High
2. Aune, 2009	RCT	Norway	Secondary	135	The Norwegian Universal Preventive Program for Social Anxiety CB	Psychologist	N/A—Professional	Secondary	SDQ	No intervention	Some concerns
3. Britton, 2014	RCT	US	Primary	210	No name M	Teacher	Yes	Primary	YSR	Active control	Some concerns
4. Carroll, 2020	QED	Australia	Secondary	715	KooLKIDS SE	Teacher	Yes	Primary	SDQ	Wait list control	Some concerns
5. Dray, 2017	RCT	Australia	Secondary	1,080	No name Multiple	Teacher	Yes	Secondary	SDQ	No intervention	High
6. Gucht, 2018	RCT	Belgium	Primary	480	No name ACT	Teacher	Yes	Secondary	YSR	No intervention	Low
7. Holen, 2012	RCT	Norway	Secondary	1,320	Zippy's Friends NLEC	Teacher	Yes	Primary	SDQ	No intervention	Low
8. Humphrey, 2016	RCT	England	Secondary	1,400	Promoting alternative thinking strategies (PATHS) Multiple	Teacher	Yes	Primary	SDQ	No intervention	Some concerns
9. Khalsa 2011	RCT	US	Secondary	825	Yoga Ed Y	Facilitator	N/A—Professional	Secondary	POMS-SF	No intervention	Some concerns
10. Kuyken, 2022	RCT	England	Secondary	400	No name (MYRIAD study) M	Teacher	Yes	Secondary	SDQ	No intervention	Some concerns
11. Lam, 2020	QED	Hong Kong	Secondary	420	Learning to BREATHE M	Psychologist	N/A—Professional	Secondary	YSR	No intervention	Low
12. Muratori, 2017	RCT	Italian	Secondary	1,440	Coping power SC	Teacher	Yes	Secondary	SDQ	No intervention	Low
13. Myles-Pallister, 2014	RCT	Australia	Secondary	480	Aussie optimism positive thinking skills program CB	Psychologist	Yes	Primary	SDQ	No intervention	Some concerns
14. Roberts, 2010	RCT	Australia	Primary	600	Aussie optimism program CB	Teacher	Yes	Primary	CBC	No intervention	High
15. Takahashi, 2020	QED	Japan	Secondary	300	No name ACT	Psychologist	N/A—Professional	Secondary	SDQ	Wait list control	Some concerns

ACT, acceptance and commitment therapy; BASC, behaviour assessment system for children; CB, cognitive behavioural; CBC, child behaviour checklist; D, developmental; DBT, dialectical behaviour therapy; M, mindfulness; N/A, not applicable; NLEC, negative life events and coping; QED, quasi experimental design; SC, socio-cognitive; SDQ, strengths and difficulties questionnaire; SE, socio-emotional learning; RCT, randomised controlled trial; US, United States; YSR, youth self report.

### Anxiety

**Figure 2:**
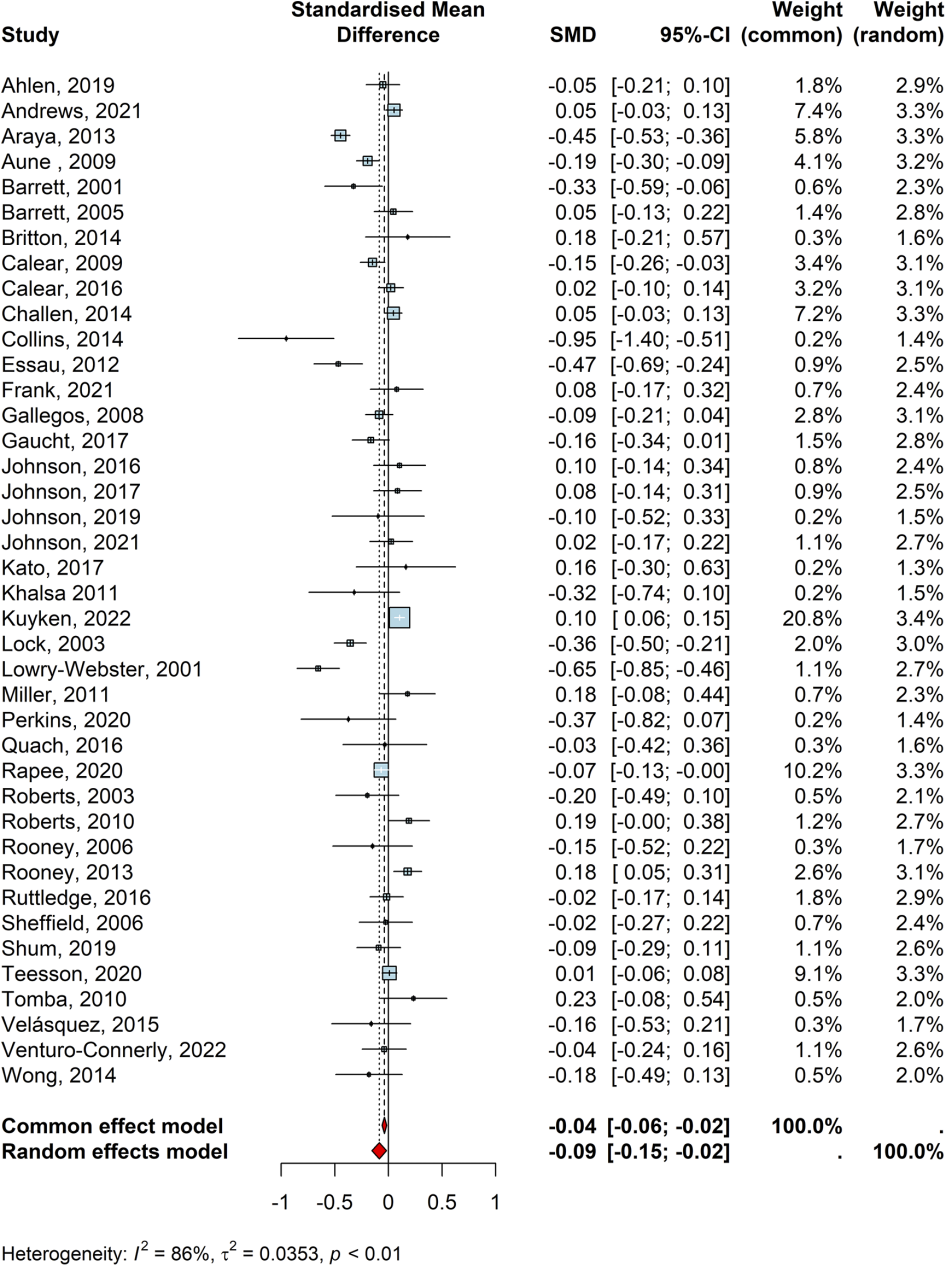
Forest plot for anxiety outcomes.

In total, 40 studies reported the efficacy of universal interventions on anxiety outcomes of children and young people (*n* = 35,559). Of these, 24 studies individually reported that universal interventions were effective in reducing anxiety outcomes, though only 10 of these were statistically significant (Table 1: Araya, 2013 “Yo, Pienso, Siento”; Aune, 2009 “The Norwegian Universal Preventive Program for Social Anxiety”; Barrett, 2001 “Friends for Children”; Calear, 2009 “MoodGYM”; Collins, 2014; “No name”; Essau, 2012 “FRIENDS”, Gaucht, 2017 “No name”; Lock, 2003 “FRIENDS”, Lowry-Webster, 2001 “FRIENDS”, and Rapee, 2020 “Friendly Schools and Cool Kids—Taking Control”). A random effect meta-analysis was conducted to pool these individual effect sizes from 40 studies which indicated a statistically significant, but small, negative effect size (d = −0.0858, CI = −0.15, −0.02, z = −2.46, *p* < .01; Figure 2). No individual studies had a driving influence (i.e., meta-influence) on the pooled effect size for the anxiety outcome and the Egger's test (t = −1.69, df = 38, *p* = 0.09) and the visual inspection of the funnel plot (S4) indicated no potential publication bias. This finding indicates that children and young people who received universal interventions were better off than those in the control groups in terms of experiencing symptoms of anxiety. However, these studies showed high heterogeneity (I2 = 85%, *τ*2 = 0.03, *p* < 0.01), hence, we conducted subgroup analyses to test potential influence of study characteristics on the pooled effect size. This revealed that the pooled effect size was moderated by certain study characteristics such as study design (Q = 4.10, *p* = 0.042), control type (Q = 9.43, *p* < 0.01), and intervention theory (Q = 24.93, *p* < 0.001). Specifically, interventions that were compared to no intervention/practice as usual were significantly more effective than those that were controlled against an active intervention group. This suggests that children and young people who received a specific universal intervention for anxiety were better off than those who received school practice as usual. Additionally, universal interventions that applied CBT principles were significantly more effective than those that applied mindfulness or other/multiple theories. Finally, interventions delivered as part of an RCT were significantly more effective than those as part of QED potentially due to the fact that RCTs better reflect intervention effects due to true randomisation and baseline equivalence.

Methodological quality, outcome type, intervention length, who delivered the intervention, or school type played no moderating role between universal interventions and anxiety outcomes Details for the subgroup analysis and funnel plot can be seen in the Supplementary Materials S4.

### Depression

**Figure 3:**
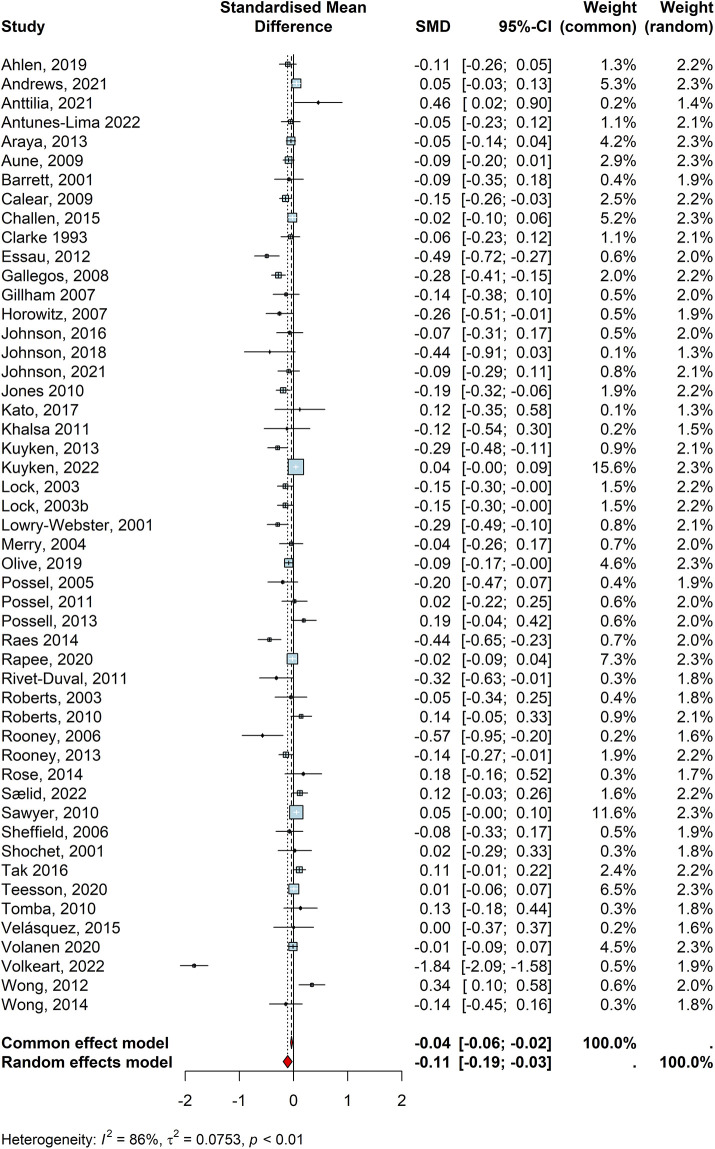
Forest plot for depression outcomes.

Overall, 50 studies reported depression outcomes for children and young people (*n* = 49,418). Of the included studies, 34 suggested that the delivered intervention reduced depression symptoms, though only 15 of these were statistically significant (Table 2: Calear, 2009 “MoodGYM”; Essau, 2012 “FRIENDS”; Gallegos, 2008 “FRIENDS for Life”, Horowitz, 2007 “No name”; Jones, 2010 “4Rs Program”; Kuyken, 2013 “MiSP programme”; Lock, 2003 “FRIENDS”;Lock, 2003b “FRIENDS”; Lowry-Webster, 2001 “FRIENDS”; Olive, 2019 “No name”; Raes, 2014 “No name”; Rivet = Duval, 2011 “RAP-A”; Rooney, 2006 “Positive Thinking Programme”; Rooney, 2013 “Positive Thinking Skills Program”; and Volkeart, 2022 “Boost Camp”. Pooling all the individual effect sizes in a random effect meta-analysis provided a negative, but small, effect size (d = −0.109, CI = −0.19, −0.03, z = −2.60, *p* < 0.013; Figure 3). All studies had an average influence on the pooled effect size for the depression outcome. This suggests that children and young people who received a specific universal intervention for depression had significantly lower rates of depressive symptoms compared to those who did not. However, the high heterogeneity (I2 = 86%, *τ*2 = 0.07, *p* < 0.01) indicated that the reported effect size may have been moderated by heterogenous study characteristics. Upon performing subgroup analyses, we found that certain study characteristics such as control type (Q = 8.26, *p* < 0.01) moderated the pooled effect size of the universal interventions on depression. Methodological quality, intervention theory, outcome type, intervention length, school type, and who delivered the intervention did not have any significant impact on the efficacy of such trials on depression outcomes. More specifically, similar to what was found for the anxiety outcome, universal interventions that delivered against practice as usual or no intervention control groups were more effective than those delivered against an active control group. That said, children and young people who received universal interventions had lower rates of depression symptoms than those who received no treatment at school. In contrast with findings for the anxiety outcome, there were no significant differences between interventions that applied CBT principles and those based on mindfulness or other/multiple theories. Finally, the visual inspection of the funnel plot and the Egger's test result (t = −2.64, df = 48, *p* < .01) also indicated a potential publication bias for the meta-analysis of studies reporting the depression outcome. Details for the subgroup analysis and funnel plot can be seen in the Supplementary Materials S5.

### Internalising problems

**Figure 4:**
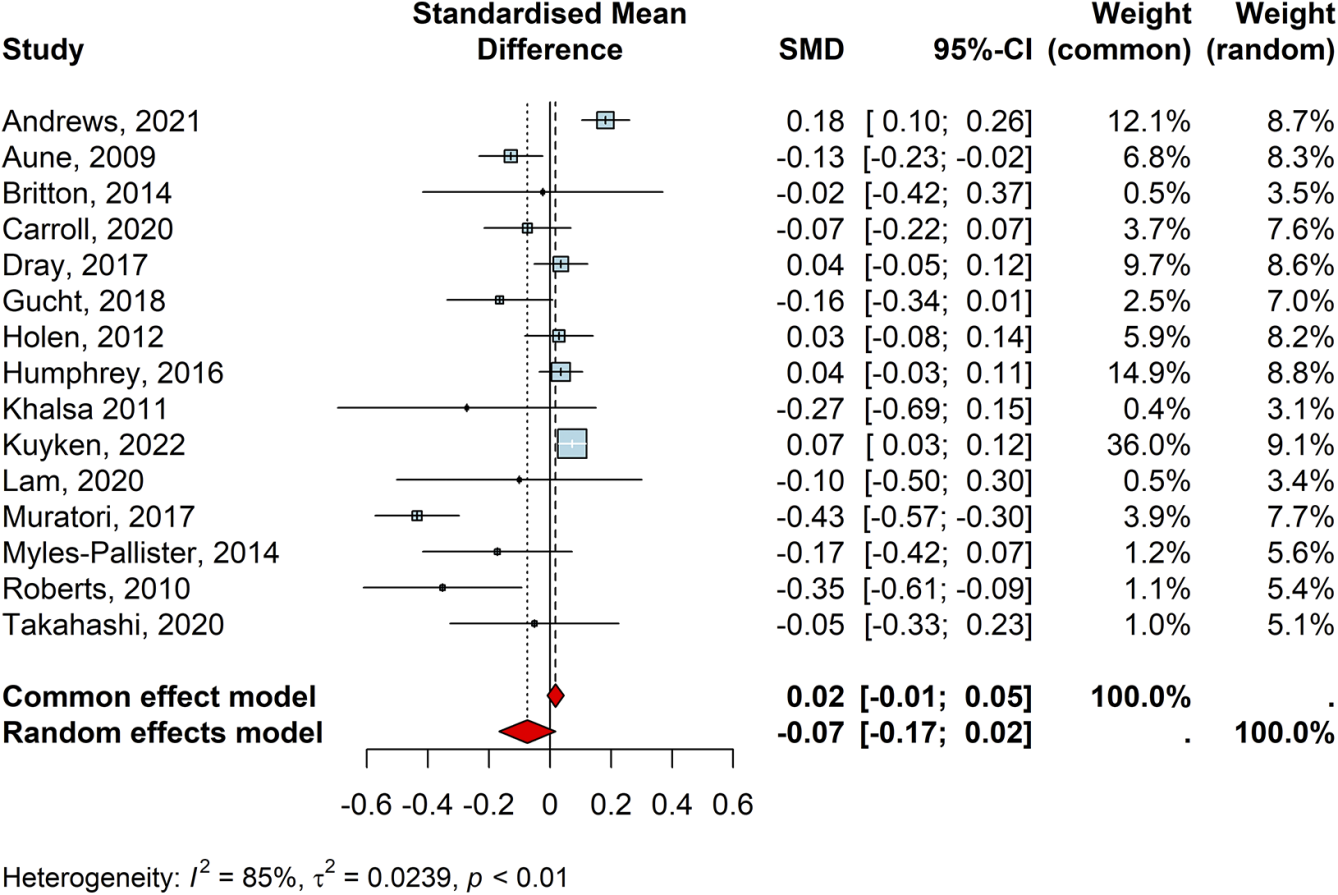
Forest plot for internalising difficulties outcomes.

There were 15 studies reporting the efficacy of universal interventions on internalising symptoms of children and young people(*n* = 21,473). Of these, 10 studies individually reported reduced rates of internalising difficulties for children and young people following a universal intervention, though only three of them were statistically significant (Table 3: Aune, 2009 “The Norwegian Universal Preventive Program for Social Anxiety”; Muratori, 2017 “Coping Power”, and Roberts, 2010 “Aussie Optimism Program”. A random effect meta-analysis pooling the individual effect sizes indicated no significant effect for the efficacy of such interventions on the internalising difficulties of pupils (d = −0.740, CI = −0.17, 0.02, z = −1.57, *p* = 0.11; I2 = 85%, *τ*2 = 0.02, Figure 4). The funnel plot and Egger's test (t = −2.55, df = 13, *p* = 0.02) showed potential publication bias for the meta-analysis of studies reporting internalising difficulties. Additionally, none of the included studies had a significant meta-influence driving the pooled effect size for the internalising difficulties outcome. Publication bias and meta-influence plot can be found in the Supplementary Materials S6. No subgroup analyses are reported as there were no significant effects of the included universal interventions on internalising outcomes.

## Discussion

We aimed to investigate the impact of universal school-based interventions on emotional outcomes taking into account limitations from previous reviews. In line with some previous meta-analyses, we found that universal school-based interventions have a statistically significant but small effect for symptoms of both anxiety and depression outcomes (19, 21). However, no such effect was found for internalising outcomes. There has been much debate in the academic discourse on the magnitude of effect sizes and the degree to which they represent whether an intervention is meaningful. Carey and colleagues (33) posit the importance of context when inferring intervention impact with small effect sizes, highlighting that at an individual level, a small effect size could translate to a perceived inconsequential change on a symptomology measure for one patient, yet at a population health level, scaling interventions with small effect sizes can have impactful change. Additionally, given the increasing prevalence rates of youth mental health difficulties, with the latest estimates showing 1 in 5 young people now have a probable mental health difficulty (34), and 1 in 3 of those do not reach out for any professional support, the need for wide-reaching, effective, preventative and early interventions are crucial.

Perhaps unsurprisingly, subgroup-analyses for both anxiety and depression interventions found that interventions that were compared with no intervention/practice as usual showed greater impact than those that were controlled against an active intervention group. This suggests that providing some level of intervention could be better than doing nothing at all. However, no treatment or practice as usual represents a “low bar” against which to judge programme effectiveness. Therefore, funders of future programmes may wish to move towards comparing studies to active controls. We also found that CBT-informed approaches were significantly more effective than those that applied mindfulness or other/multiple approaches for anxiety outcomes. However, intervention type did not moderate depressive symptoms. Mindfulness has rapidly gained prominence in school curriculums in recent years, yet results from this review suggest that optimising CBT programmes over other modalities would be beneficial to prevent, or reduce, anxiety symptoms. As such, schools and public health officials should critically consider underlying modalities before implementing universal anxiety programmes. Contrary to the most recent review on this topic (14), we found that effect sizes for universal interventions were not moderated by whether interventions were delivered in primary or secondary schools. This could mean that primary schools may be an important setting to first deliver universal interventions to help prevent mental health difficulties, particularly as prevalence of emotional difficulties increases with the onset of adolescence (35). Lastly, we also found that the intervention deliverer did not moderate anxiety and depressive outcomes which also aligns with previous research (14). In conjunction with the finding that intervention length did not moderate symptomology, this suggests that there are a variety of different programmes that may have efficacy, and schools should have the flexibility to select and fully deliver universal programme that suit them in both time commitment and staff who feel able to deliver such programmes. In light of these findings on effectiveness, it is possible that when implementing universal interventions, sufficient attention should also be placed on acceptability and satisfaction so children will be more likely to engage.

A number of limitations should be acknowledged in relation to the current paper. First, whilst different databases were searched and experts consulted, it is still possible that some studies may have been missed. This may be particularly true when it comes to internalising difficulties as the term is not universally applied or where it has been measured as a secondary outcome and not reported in the title or abstract. However, to try and combat this, other similar terms, such as broadly defined emotional difficulties, were included. However, this means that different, but similar, symptom profiles may have been grouped together, so caution is advised when interpreting these results. Second, we were only able to separate sub-group analyses into a maximum of three groups to minimise false positives. This resulted in the merging of some categories which could distort or hide the impact of some intervention characteristics. Third, depression and internalising problems showed potential publication bias for the meta-analysis of studies, so caution is suggested when interpreting these results. Future meta-analyses and researcher guidelines may wish to consider how these limitations can be addressed when investigating universal interventions for pupils mental health, as well as explore the sustained impact of said interventions on such symptom profiles over time. Additionally, given that implementation factors, such as fidelity and dosage are known to impact outcomes (36), future research may wish to account for implementation factors when conducting such meta-analyses.

Notwithstanding these limitations, the current findings lend weight to the argument that universal programmes aimed at tackling depression and anxiety can be beneficial. Given the national and global trends showing incremental increases in rates of anxiety and depression difficulties in adolescents and the high numbers of individuals who do not reach out for formal support to health services, such programmes can play a modest but significant role in improving population level mental health for young people. However, findings also indicate that not all universal programmes are equal. While differences between impacts for interventions focused on different practices (e.g., mindfulness, CBT) warrant replication, they do emphasise the importance of providing clear evidence-based guidance to schools around effective and evidence-based practice to ensure time and resource is not wasted on ineffective approaches.

## Data Availability

The raw data supporting the conclusions of this article will be made available by the authors, without undue reservation.

## References

[1] Bor W Dean AJ Najman J Hayatbakhsh R. Are child and adolescent mental health problems increasing in the 21st century? A systematic review. Aust N Z J Psychiatry. (2014) 48(7):606–16. 10.1177/0004867414533834 24829198

[2] Patalay P Gage SH. Changes in millennial adolescent mental health and health-related behaviours over 10 years: a population cohort comparison study. Int J Epidemiol. (2019) 48(5):1650–64. 10.1093/ije/dyz006 30815691 PMC6904321

[3] World Health Organisation. Global accelerated action for the health of adolescents (AA-HA!): guidance to support country implementation. Geneva. (2017). Available at: https://iris.who.int/bitstream/handle/10665/255415/9789241512343-eng.pdf?sequence=1 (Accessed October, 07, 2024).

[4] Nguyen T Hellebuyck M Halpern M Fritze D. The State of Mental Health in America. (2018).

[5] Naicker K Galambos NL Zeng Y Senthilselvan A Colman I. Social, demographic, and health outcomes in the 10 years following adolescent depression. J Adolesc Health. (2013) 52(5):533–8. 10.1016/j.jadohealth.2012.12.016 23499382

[6] Essau CA Lewinsohn PM Olaya B Seeley JR. Anxiety disorders in adolescents and psychosocial outcomes at age 30. J Affect Disord. (2014) 163:125–32. 10.1016/j.jad.2013.12.033 24456837 PMC4028371

[7] Fazel M Patel V Thomas S Tol W. Mental health interventions in schools in low-income and middle-income countries. Lancet Psychiatry. (2014) 1(5):388–98. 10.1016/S2215-0366(14)70357-8 26361001

[8] Fazel M Hoagwood K Stephan S Ford T. Mental health interventions in schools in high-income countries. Lancet Psychiatry. (2014) 1(5):377–87. 10.1016/S2215-0366(14)70312-8 26114092 PMC4477835

[9] Caan W Cassidy J Coverdale G Ha MA Nicholson W Rao M. The value of using schools as community assets for health. Public Health. (2015) 129(1):3–16. 10.1016/j.puhe.2014.10.006 25481543

[10] Department of Health and Department for Education. Transforming Children and Young People’s Mental Health Provision: A Green Paper. London: Department of Health and Department for Education (2017).

[11] Memon A Taylor K Mohebati LM Sundin J Cooper M Scanlon T Perceived barriers to accessing mental health services among black and minority ethnic (BME) communities: a qualitative study in southeast England. BMJ Open. (2016) 6(11):e012337. 10.1136/bmjopen-2016-012337 27852712 PMC5128839

[12] Weist MD Evans SW. Expanded school mental health: challenges and opportunities in an emerging field. J Youth Adolesc. (2005) 34(1):3–6. 10.1007/s10964-005-1330-2

[13] Shoshani A Steinmetz S. Positive psychology at school: a school-based intervention to promote adolescents’ mental health and well-being. J Happiness Stud. (2014) 15(6):1289–311. 10.1007/s10902-013-9476-1

[14] Caldwell DM Davies SR Hetrick SE Palmer JC Caro P López-López JA School-based interventions to prevent anxiety and depression in children and young people: a systematic review and network meta-analysis. Lancet Psychiatry. (2019) 6(12):1011–20. 10.1016/S2215-0366(19)30403-1 31734106 PMC7029281

[15] Vostanis P Humphrey N Fitzgerald N Deighton J Wolpert M. How do schools promote emotional well-being among their pupils? Findings from a national scoping survey of mental health provision in English schools. Child Adolesc Ment Health. (2013) 18(3):151–7. 10.1111/j.1475-3588.2012.00677.x 32847252

[16] Hayes D Moore A Stapley E Humphrey N Mansfield R Santos J School-based intervention study examining approaches for well-being and mental health literacy of pupils in year 9 in England: study protocol for a multischool, parallel group cluster randomised controlled trial (AWARE). BMJ Open. (2019) 9(8):e029044. 10.1136/bmjopen-2019-029044 31481370 PMC6731836

[17] Hayes D Moore A Stapley E Humphrey N Mansfield R Santos J Promoting mental health and well-being in schools: examining mindfulness, relaxation and strategies for safety and well-being in English primary and secondary schools—study protocol for a multi-school, cluster randomised controlled trial (INSPIRE). Trials. (2023) 24(1):220. 10.1186/s13063-023-07238-8 36959662 PMC10034911

[18] McLaughlin KA. The public health impact of major depression: a call for interdisciplinary prevention efforts. Prev Sci. (2011) 12(4):361–71. 10.1007/s11121-011-0231-8 21732121 PMC3219837

[19] Stockings EA Degenhardt L Dobbins T Lee YY Erskine HE Whiteford HA Preventing depression and anxiety in young people: a review of the joint efficacy of universal, selective and indicated prevention. Psychol Med. (2016) 46(1):11–26. 10.1017/S0033291715001725 26315536

[20] Johnstone KM Kemps E Chen J. A meta-analysis of universal school-based prevention programs for anxiety and depression in children. Clin Child Fam Psychol Rev. (2018) 21(4):466–81. 10.1007/s10567-018-0266-5 30105480

[21] Werner-Seidler A Perry Y Calear AL Newby JM Christensen H. School-based depression and anxiety prevention programs for young people: a systematic review and meta-analysis. Clin Psychol Rev. (2017) 51:30–47. 10.1016/j.cpr.2016.10.005 27821267

[22] Caldwell DM Davies SR Thorn JC Palmer JC Caro P Hetrick SE School-based interventions to prevent anxiety, depression and conduct disorder in children and young people: a systematic review and network meta-analysis. Public Health Research. (2021) 9(8):1–284. 10.3310/phr09080 34347403

[23] Shreffler J Huecker MR. Type I and Type II Errors and Statistical Power. (2024). 32491462

[24] Fazel M Kohrt BA. Prevention versus intervention in school mental health. Lancet Psychiatry. (2019) 6(12):969–71. 10.1016/S2215-0366(19)30440-7 31734107

[25] Torgerson CJ Porthouse J Brooks G. A systematic review and meta-analysis of randomised controlled trials evaluating interventions in adult literacy and numeracy. J Res Read. (2003) 26(3):234–55. 10.1111/1467-9817.00200

[26] Sterne JAC Savović J Page MJ Elbers RG Blencowe NS Boutron I Rob 2: a revised tool for assessing risk of bias in randomised trials. Br Med J. (2019) 366:l4898. 10.1136/bmj.l4898 31462531

[27] Sterne JA Hernán MA Reeves BC Savović J Berkman ND Viswanathan M ROBINS-I: a tool for assessing risk of bias in non-randomised studies of interventions. Br Med J. (2016) 355:i4919. 10.1136/bmj.i4919 27733354 PMC5062054

[28] Deniz E Francis G Torgerson C Toseeb U. Parent-mediated play-based interventions to improve social communication and language skills of preschool autistic children: a systematic review and meta-analysis. Rev J Autism Dev Disord. (2024). 10.1007/s40489-024-00463-0 35969530 PMC9377609

[29] Burke JF Sussman JB Kent DM Hayward RA. Three simple rules to ensure reasonably credible subgroup analyses. Br Med J. (2015) 351:h5651. 10.1136/bmj.h5651 26537915 PMC4632208

[30] Spence SH. Spence children’s anxiety scale. APA PsycTests. (1997). 10.1037/t10518-000

[31] Kovacs M. The Children’s Depression Inventory (CDI) Manual North Tanawanda. New York: Multi-Health Systems (1992).

[32] Goodman R. The strengths and difficulties questionnaire: a research note. J Child Psychol Psychiatry. (1997) 38(5):581–6. 10.1111/j.1469-7610.1997.tb01545.x 9255702

[33] Carey EG Ridler I Ford TJ Stringaris A. Editorial perspective: when is a ’small effect’ actually large and impactful? J Child Psychol Psychiatry. (2023) 64(11):1643–7. 10.1111/jcpp.13817 37226639

[34] NHS Digital. Mental Health of Children and Young People in England, 2023 - Wave 4 Follow up to the 2017 Survey. London: NHS Digital (2023). Available at: https://digital.nhs.uk/data-and-information/publications/statistical/mental-health-of-children-and-young-people-in-england/2023-wave-4-follow-up# (Accessed February 16, 2024).

[35] World Health Organisation. Mental Health of Adolescents. Geneva: World Health Organization (2021). Available at: https://www.who.int/news-room/fact-sheets/detail/adolescent-mental-health (Accessed Februay 16, 2024).

[36] Durlak JA DuPre EP. Implementation matters: a review of research on the influence of implementation on program outcomes and the factors affecting implementation. Am J Community Psychol. (2008) 41(3-4):327–50. 10.1007/s10464-008-9165-0 18322790

[37] Moher D Liberati A Tetzlaff J Altman DG. Preferred reporting items for systematic reviews and meta-analyses: the PRISMA statement. Br Med J. (2009) 339:b2535. 10.1136/bmj.b2535 19622551 PMC2714657

