# Supplementary material for: Universal, school-based, interventions to improve emotional outcomes in children and young people: a systematic review and meta-analysis
Source: Front Child Adolesc Psychiatry. 2025 Jun 2;4:1526840. doi: 10.3389/frcha.2025.1526840 (PMC12171270; doi:10.3389/frcha.2025.1526840)
Supplement: Supplementary file 1 [file Supplementaryfile1.docx]

**Supplementary information**

*S0 PICO criteria*

| Patient/population, | Pupils aged between 8-18 years old |
| --- | --- |
| Intervention, | Universal, school-based interventions |
| Comparison | Any control group (active, wait-list or usual practice) |
| Outcomes | Anxiety, depression or internalising difficulties |

*S1 Search strategy.*

Title, abstract and keyword search

Concept 1: Children and young people

1. adolescen*
2. or teen*
3. or youth*
4. or child*
5. or minor*
6. or 'young people*'
7. or 'young person*'
8. or student*
9. or pupil*
10. or pediatric*
11. or paediatric*
12. 1 or 2 or 3 or 4 or 5 or 6 or 7 or 8 or 9 or 10 or 11

Concept 2: Emotional difficulties

1. Depress*
2. Low mood
3. Anxiety*
4. Anxious
5. Worry
6. Emotional diff*
7. Emotional disorder*
8. Emotional problem*
9. Emotional illness*
10. Emotional health
11. Emotional symptom*
12. Internali*
13. Mental health
14. Mental illness
15. Mental disorder
16. Emotional learning
17. Emotional skills
18. Emotional distress
19. Emotional development
20. Emotional wellbeing
21. Emotional outcome*
22. 13 or 14 or 15 or 16 or 17 or 18 or 19 or 20 or 21 or 22 or 23 or 24 or 25 pr 26 or 27 or 28 or 29 or 30 or 31 or 32 or 33

Concept 3: Comparator

1. RCT
2. Random*
3. Control*
4. Synthes*
5. Meta*
6. Systematic
7. Review
8. Usual provision
9. 35 or 36 or 37 or 38 or 39 or 40 or 41 or 42

Concept 4: School

1. *School
2. School*
3. Class*
4. School-based
5. School based
6. 44 or 45 or 46 or 47 or 48

Concept 5: Universal

1. Universal
2. Whole school
3. Whole-school
4. Whole class
5. Whole-class
6. School-wide
7. School wide
8. Multi-year
9. System wide
10. 50 or 51 or 52 or 53 or 54 or 55 or 56 or 57 or 58
11. 12 and 34 and 43 and 49 and 59

*S2: Definitions for examined constructs*

Internalising difficulties: difficulties associated with high levels of negative affectivity, which may include depressive disorders and anxiety disorders, but also other broader difficulties such as feeling worried low. This may be measures used subscales, such as the SDQ internalising subscale

Anxiety symptoms: symptoms associated with anxiety and anxiety disorders (e.g.generalised anxiety). This may be measures using questionnaires such as the SCAS.

Depression symptoms: symptoms associated with depression and depressive disorders (e.g. Feeling empty and hopeless). This may be measures using questionnaires such as the BDI.

*S3: Data extraction table headings*

| Author | Design | Country | Outcome | Length | Theory | Deliverer | Training | School | Measure | Control Comparator | Control  N | Control  M | Control SD | Intervention  N | Intervention  M | Intervention SD | ROB |
| --- | --- | --- | --- | --- | --- | --- | --- | --- | --- | --- | --- | --- | --- | --- | --- | --- | --- |
|  |  |  |  |  |  |  |  |  |  |  |  |  |  |  |  |  |  |

*S4a: Funnel plot for anxiety outcomes*


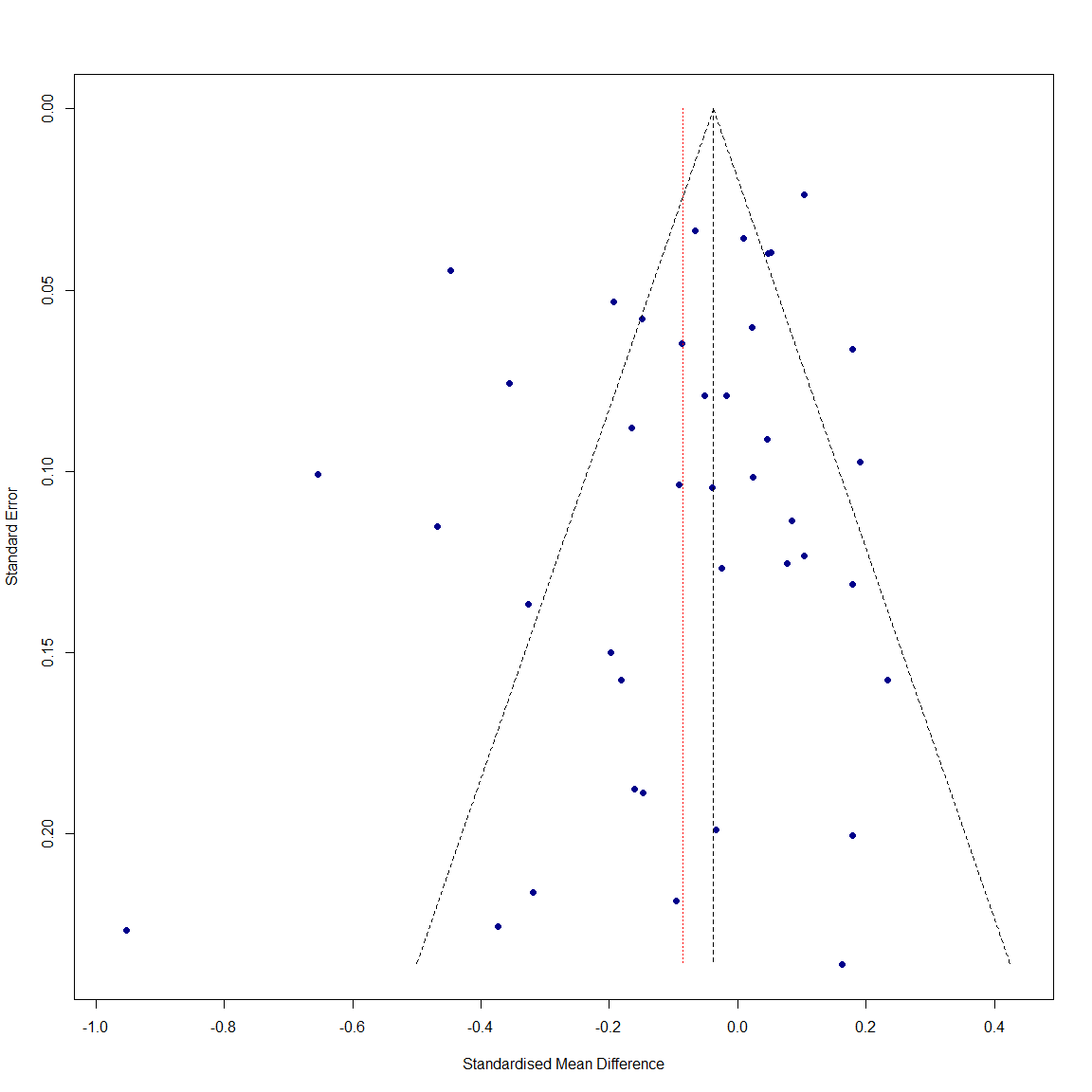


*S4b Forest Plot for Influence Analysis – anxiety outcomes*


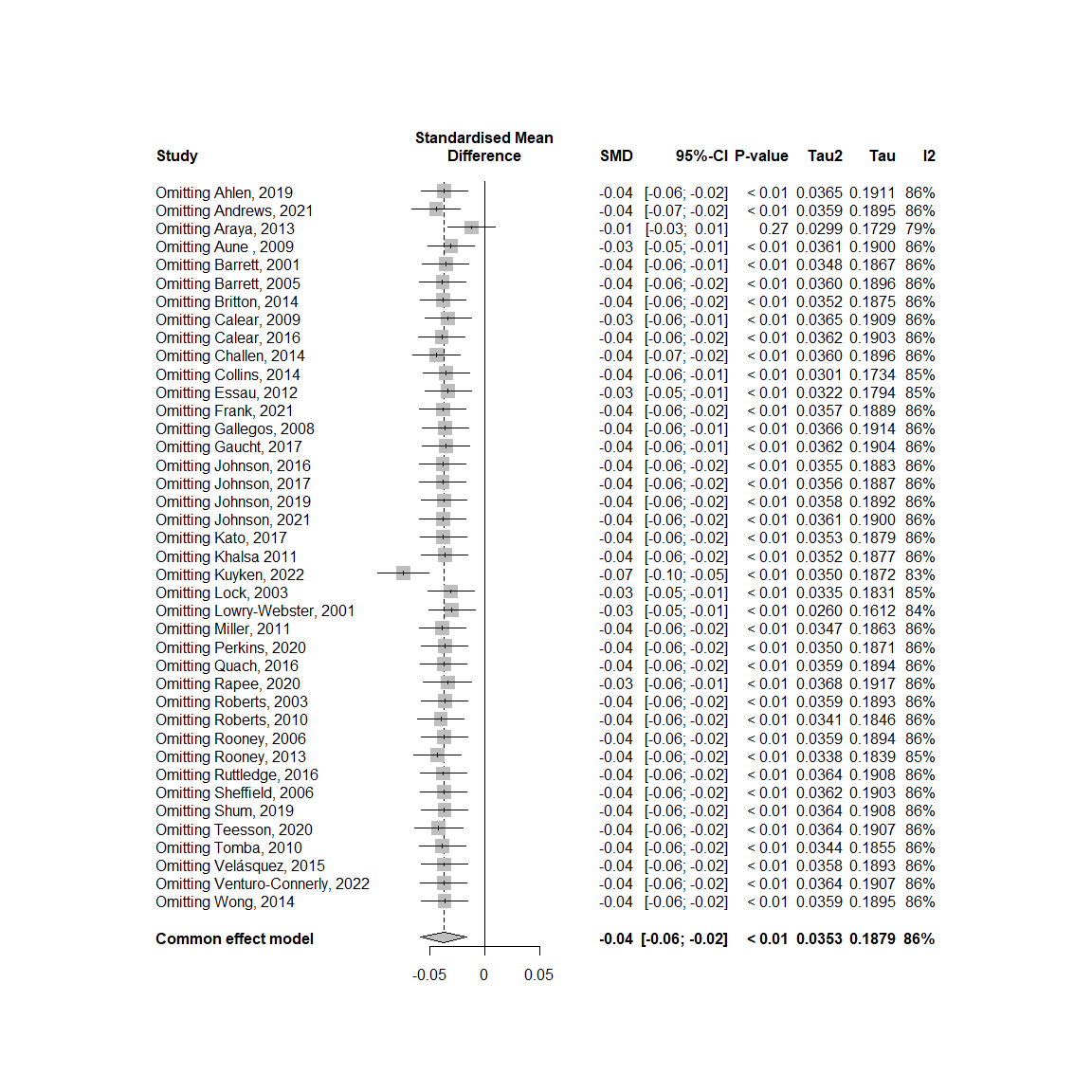


*S4c Subgroup analysis comparisons*

| Category | Subgroups | Rationale |
| --- | --- | --- |
| Design | RCT versus QED design | These were the two design types included in the review. There may be differences between QED and RCT designs, as QEDs may not have groups equal on important characterises due to a lack of randomisation |
| School type | Primary versus Secondary school | These were the two design types included in the review. Previous reviews suggest that there may be differences in outcomes included in this study for pupils in primary compared to secondary schools |
| Length | <500 mins versus 500-750 mins versus >750 mins | Length was split into tertiles. 1 SD below the mean, between -1SD and +1SD, and >+1SD above the mean |
| Intervention theory | CBT versus Mindfulness versus Other | CBT, followed by Mindfulness reflected the most common interventions being used in universal school-based programmes. The wide variety in other intervention theory after this was collapsed into the other category. |
| Intervention deliverer | Teacher versus Psychologist versus Other | Teachers, followed by Psychologists reflected the most common intervention deliverers when employing universal school-based programmes. Other individuals included facilitators who had expertise in a specific topic (e.g. Yoga), but had no specific teaching or mental health expertise, lay individuals, as well as self-directed and unguided programmes. |
| Control group | Active versus No control | There are likely to be differences in effect size as to whether pupils in the control group receive another intervention or no support. No intervention includes pupils being on a wait list control, receiving no intervention, as well as treatment as usual. |
| Outcome | Primary versus Secondary | There were 2 outcomes included in this review. There may be differences when the intervention is aimed at the primary outcome (main intended effect) versus secondary outcomes. |
| Risk or bias assessment | High versus some concerns versus low | There were 3 ROB categories included in this review. There may be differences when the ROB is high versus low |

*S5a: Funnel plot for depression outcomes*


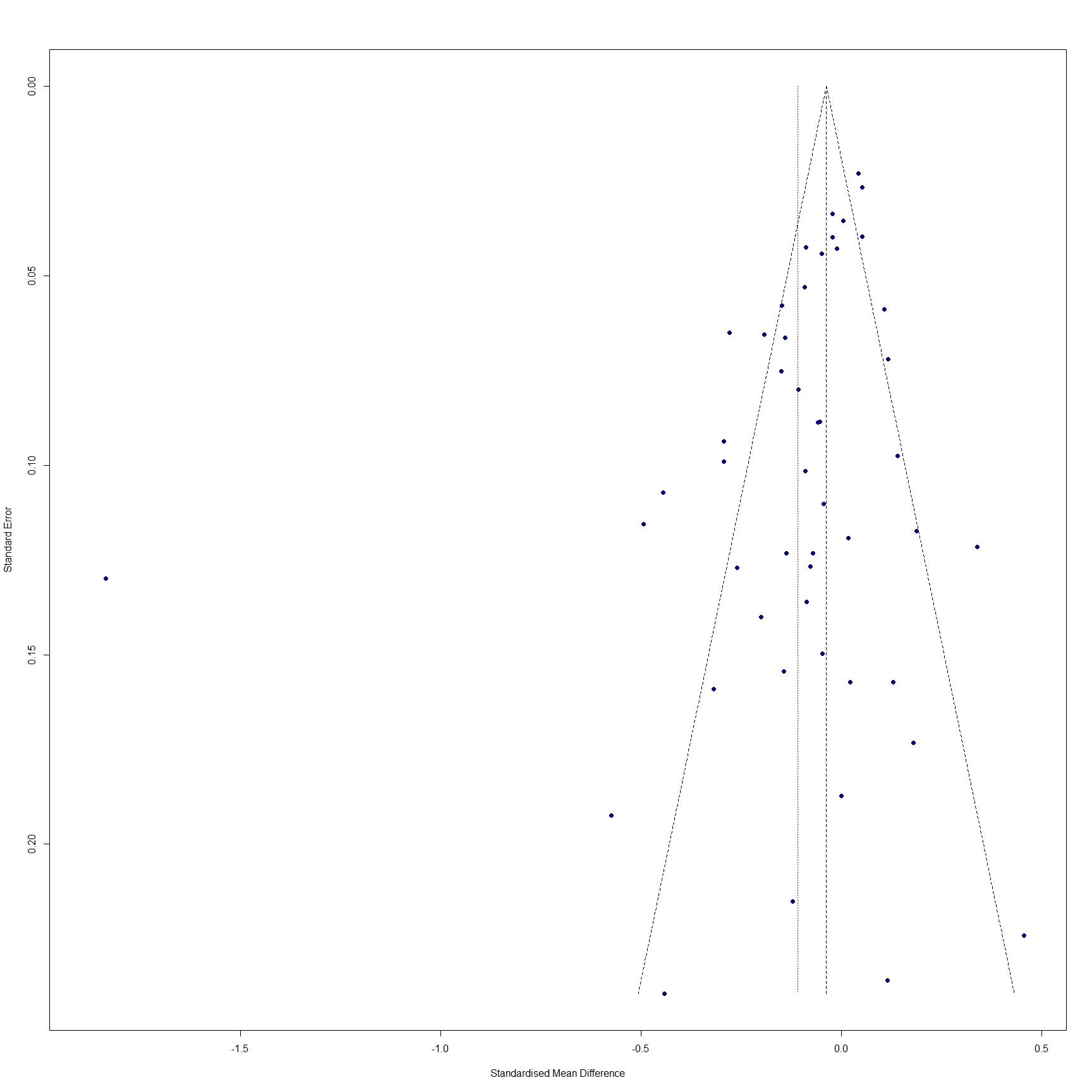


*S5b Forest Plot for Influence Analysis – depression outcomes*
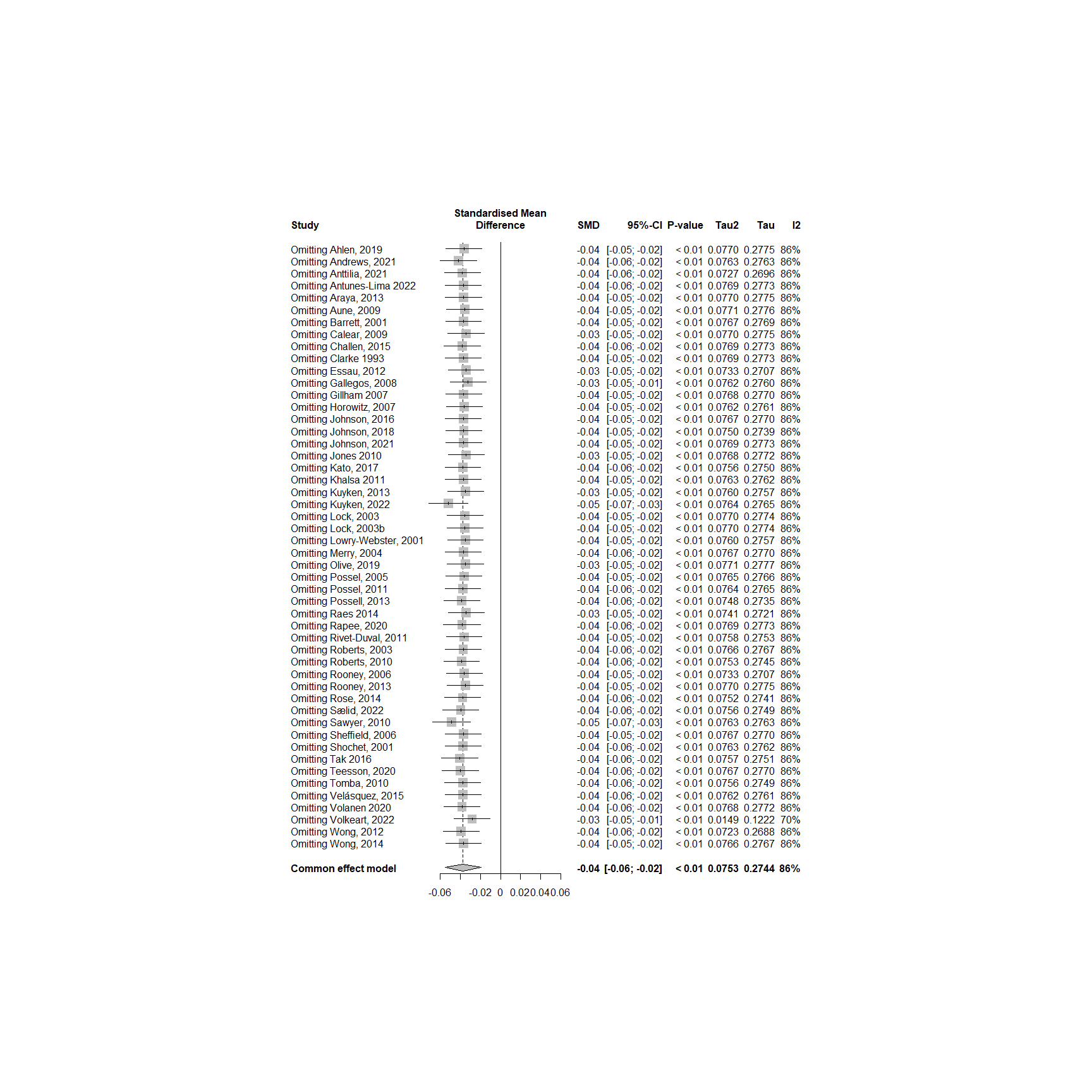


*S6a: Forest Plot for Internalising Difficulties Outcomes*


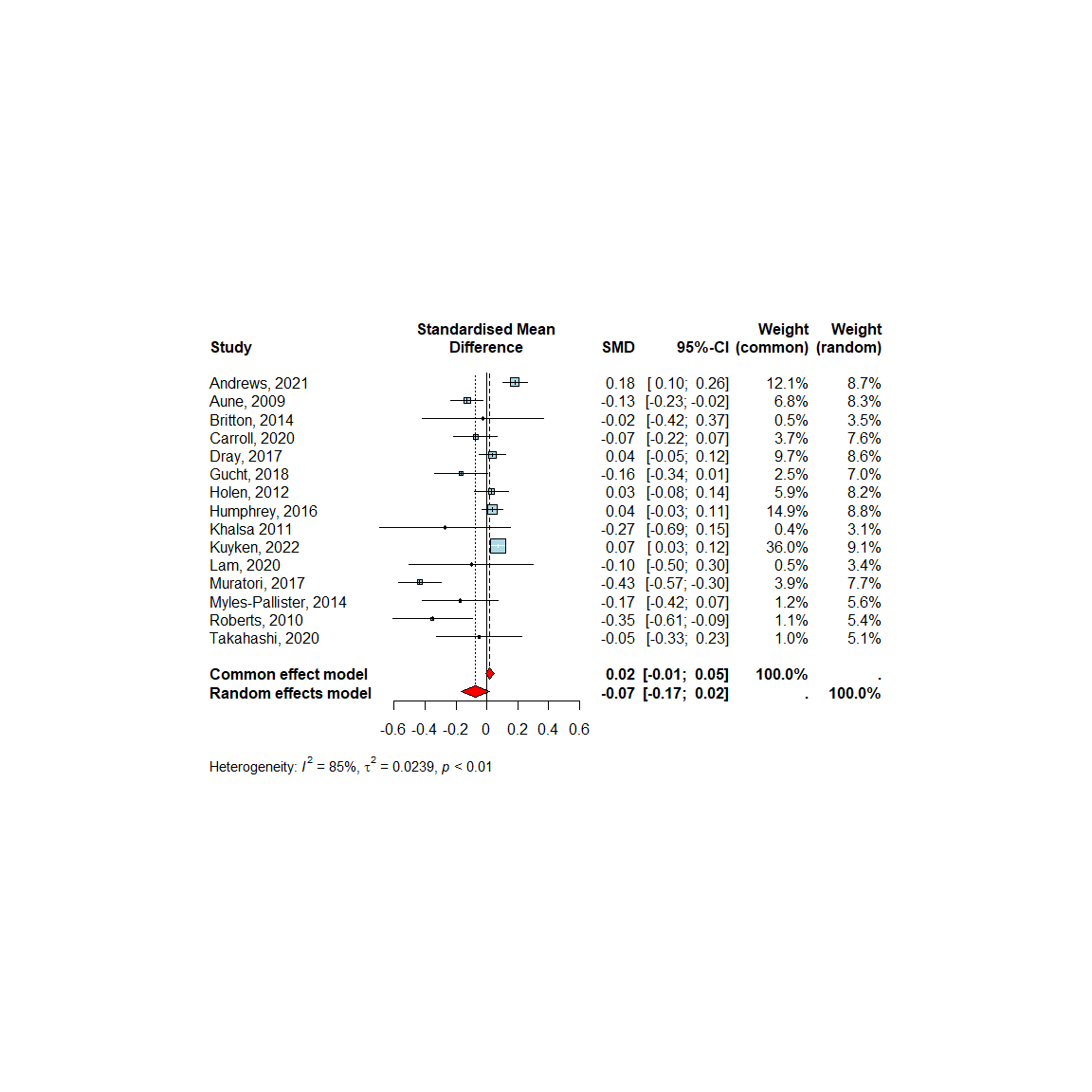


*S6b: Funnel plot for internalising outcomes*


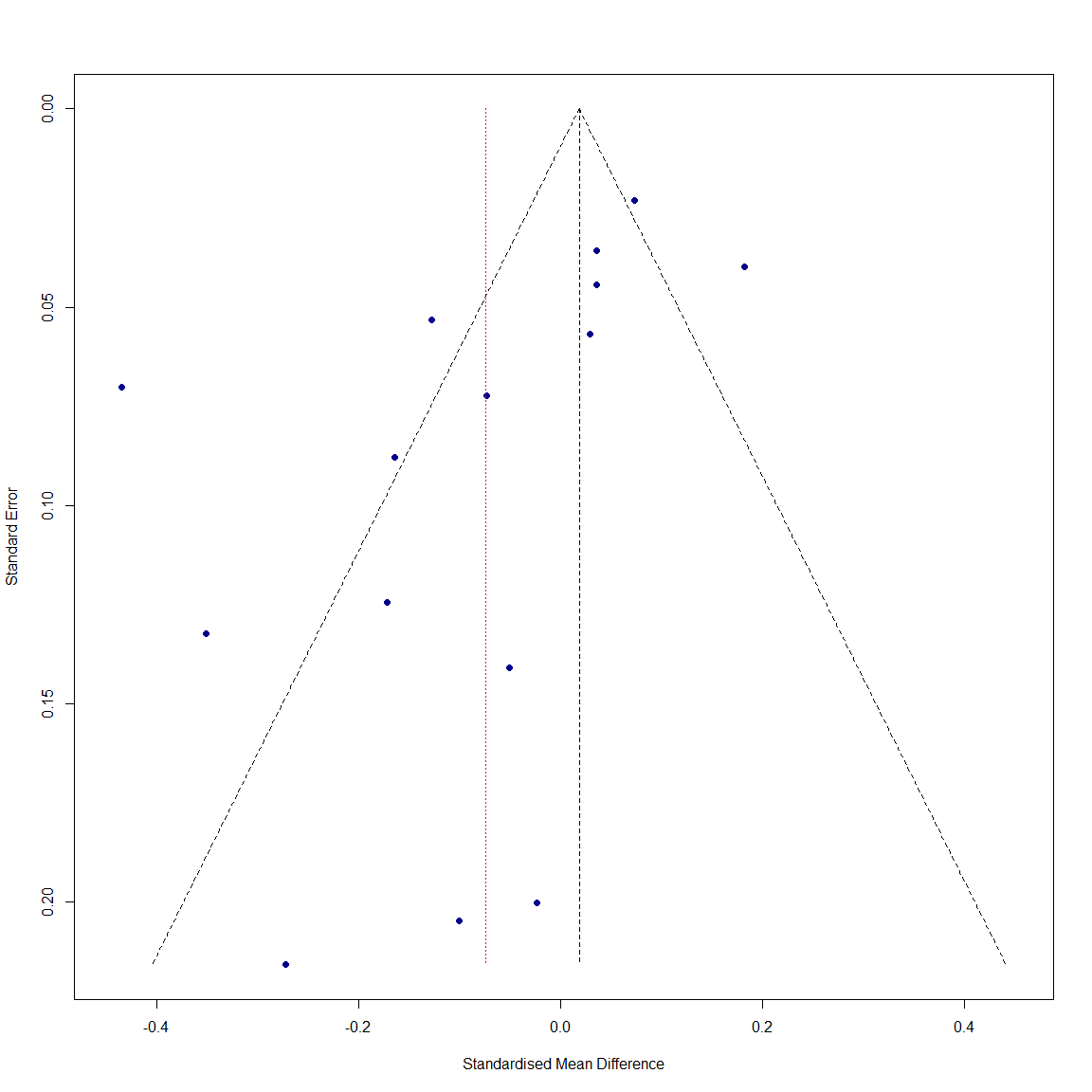


*S6c Forest Plot for Influence Analysis – internalising outcomes*
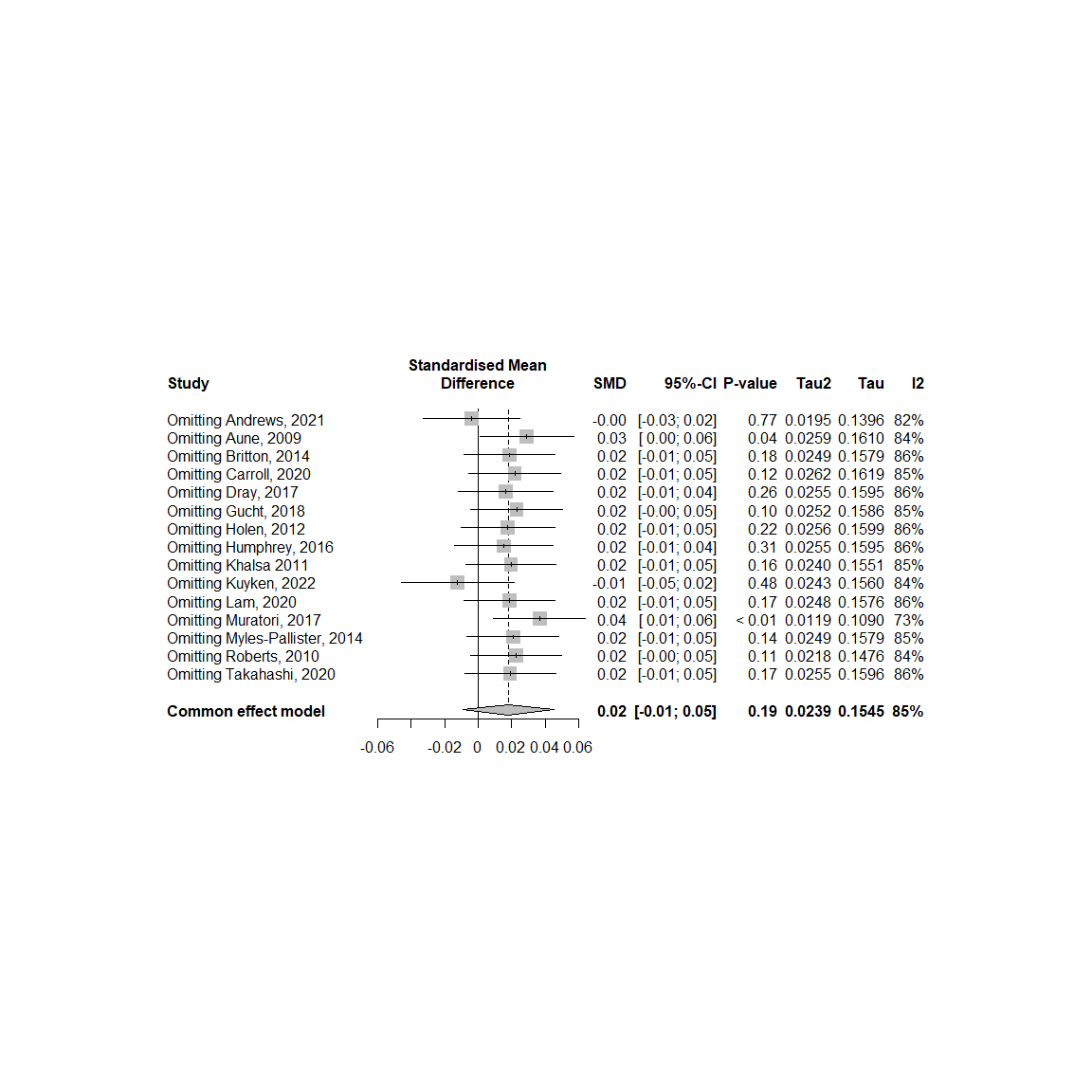


| **Section and Topic** | **Item #** | **Checklist item** | **Location where item is reported** |
| --- | --- | --- | --- |
| **TITLE** | | |  |
| Title | 1 | Identify the report as a systematic review. | 1 |
| **ABSTRACT** | | |  |
| Abstract | 2 | See the PRISMA 2020 for Abstracts checklist. | 1 |
| **INTRODUCTION** | | |  |
| Rationale | 3 | Describe the rationale for the review in the context of existing knowledge. | 2 |
| Objectives | 4 | Provide an explicit statement of the objective(s) or question(s) the review addresses. | 2-3 |
| **METHODS** | | |  |
| Eligibility criteria | 5 | Specify the inclusion and exclusion criteria for the review and how studies were grouped for the syntheses. | S0 |
| Information sources | 6 | Specify all databases, registers, websites, organisations, reference lists and other sources searched or consulted to identify studies. Specify the date when each source was last searched or consulted. | 3 |
| Search strategy | 7 | Present the full search strategies for all databases, registers and websites, including any filters and limits used. | S1 |
| Selection process | 8 | Specify the methods used to decide whether a study met the inclusion criteria of the review, including how many reviewers screened each record and each report retrieved, whether they worked independently, and if applicable, details of automation tools used in the process. | S2, 3 |
| Data collection process | 9 | Specify the methods used to collect data from reports, including how many reviewers collected data from each report, whether they worked independently, any processes for obtaining or confirming data from study investigators, and if applicable, details of automation tools used in the process. | 3 |
| Data items | 10a | List and define all outcomes for which data were sought. Specify whether all results that were compatible with each outcome domain in each study were sought (e.g. for all measures, time points, analyses), and if not, the methods used to decide which results to collect. | S1 |
|  | 10b | List and define all other variables for which data were sought (e.g. participant and intervention characteristics, funding sources). Describe any assumptions made about any missing or unclear information. | S3 |
| Study risk of bias assessment | 11 | Specify the methods used to assess risk of bias in the included studies, including details of the tool(s) used, how many reviewers assessed each study and whether they worked independently, and if applicable, details of automation tools used in the process. | 3 |
| Effect measures | 12 | Specify for each outcome the effect measure(s) (e.g. risk ratio, mean difference) used in the synthesis or presentation of results. | 3 |
| Synthesis methods | 13a | Describe the processes used to decide which studies were eligible for each synthesis (e.g. tabulating the study intervention characteristics and comparing against the planned groups for each synthesis (item #5)). | 3 |
|  | 13b | Describe any methods required to prepare the data for presentation or synthesis, such as handling of missing summary statistics, or data conversions. | 3 |
|  | 13c | Describe any methods used to tabulate or visually display results of individual studies and syntheses. | N/A |
|  | 13d | Describe any methods used to synthesize results and provide a rationale for the choice(s). If meta-analysis was performed, describe the model(s), method(s) to identify the presence and extent of statistical heterogeneity, and software package(s) used. | 3, 5 |
|  | 13e | Describe any methods used to explore possible causes of heterogeneity among study results (e.g. subgroup analysis, meta-regression). | 3, 5 |
|  | 13f | Describe any sensitivity analyses conducted to assess robustness of the synthesized results. | 3, 5 |
| Reporting bias assessment | 14 | Describe any methods used to assess risk of bias due to missing results in a synthesis (arising from reporting biases). | 3, 5 |
| Certainty assessment | 15 | Describe any methods used to assess certainty (or confidence) in the body of evidence for an outcome. | 3, 5 |
| **RESULTS** | | |  |
| Study selection | 16a | Describe the results of the search and selection process, from the number of records identified in the search to the number of studies included in the review, ideally using a flow diagram. | Fig 1 |
|  | 16b | Cite studies that might appear to meet the inclusion criteria, but which were excluded, and explain why they were excluded. | Fig 1 |
| Study characteristics | 17 | Cite each included study and present its characteristics. | 4-5 |
| Risk of bias in studies | 18 | Present assessments of risk of bias for each included study. | Tables 1-3 |
| Results of individual studies | 19 | For all outcomes, present, for each study: (a) summary statistics for each group (where appropriate) and (b) an effect estimate and its precision (e.g. confidence/credible interval), ideally using structured tables or plots. | Fig 2-4, 5, 13 |
| Results of syntheses | 20a | For each synthesis, briefly summarise the characteristics and risk of bias among contributing studies. | 5, 13, S4-6 |
|  | 20b | Present results of all statistical syntheses conducted. If meta-analysis was done, present for each the summary estimate and its precision (e.g. confidence/credible interval) and measures of statistical heterogeneity. If comparing groups, describe the direction of the effect. | 5, 13, S4-6 |
|  | 20c | Present results of all investigations of possible causes of heterogeneity among study results. | 5, 13, S4-6 |
|  | 20d | Present results of all sensitivity analyses conducted to assess the robustness of the synthesized results. | 5, 13, S4-6 |
| Reporting biases | 21 | Present assessments of risk of bias due to missing results (arising from reporting biases) for each synthesis assessed. | 5, 13, S4-6 |
| Certainty of evidence | 22 | Present assessments of certainty (or confidence) in the body of evidence for each outcome assessed. | 5, 13, S4-6 |
| **DISCUSSION** | | |  |
| Discussion | 23a | Provide a general interpretation of the results in the context of other evidence. | 14 |
|  | 23b | Discuss any limitations of the evidence included in the review. | 15 |
|  | 23c | Discuss any limitations of the review processes used. | 15 |
|  | 23d | Discuss implications of the results for practice, policy, and future research. | 15 |
| **OTHER INFORMATION** | | |  |
| Registration and protocol | 24a | Provide registration information for the review, including register name and registration number, or state that the review was not registered. | 01 |
|  | 24b | Indicate where the review protocol can be accessed, or state that a protocol was not prepared. | 01 |
|  | 24c | Describe and explain any amendments to information provided at registration or in the protocol. | - |
| Support | 25 | Describe sources of financial or non-financial support for the review, and the role of the funders or sponsors in the review. | 03 |
| Competing interests | 26 | Declare any competing interests of review authors. | 15 |
| Availability of data, code and other materials | 27 | Report which of the following are publicly available and where they can be found: template data collection forms; data extracted from included studies; data used for all analyses; analytic code; any other materials used in the review. | Supplementary information |

*From:*  Page MJ, McKenzie JE, Bossuyt PM, Boutron I, Hoffmann TC, Mulrow CD, et al. The PRISMA 2020 statement: an updated guideline for reporting systematic reviews. BMJ 2021;372:n71. doi: 10.1136/bmj.n71. This work is licensed under CC BY 4.0. To view a copy of this license, visit <https://creativecommons.org/licenses/by/4.0/>
